# A Model of Epigenetic Inheritance Accounts for Unexpected Adaptation to Unforeseen Challenges

**DOI:** 10.1002/advs.202414297

**Published:** 2025-03-18

**Authors:** Dino Osmanović, Yitzhak Rabin, Yoav Soen

**Affiliations:** ^1^ Department of Mechanical and Aerospace Engineering University of California Los Angeles Los Angeles CA 90095 USA; ^2^ Department of Physics Bar‐Ilan University Ramat Gan 5290002 Israel; ^3^ Department of Biomolecular Sciences Weizmann Institute of Science Rehovot 7610001 Israel

**Keywords:** ecology, epigenetics, mathematical biology, population dynamics

## Abstract

Accumulated evidence of transgenerational inheritance of epigenetic and symbiotic changes raises fundamental questions about the possible types, significance and duration of impacts on the population, as well as whether, and under which conditions, the inheritance of non‐genetic changes confers long‐term advantage to the population. To address these questions, a population epigenetics model of individuals undergoing stochastic changes and/or induced responses that are transmitted to the offspringis introduced. Potentially adaptive and maladaptive responses are represented, respectively, by environmentally driven changes that reduce and increase the selective pressure. Analytic solutions in a simplified case of populations that are exposed to either periodic or progressively deteriorating environments shows that acquisition and transmission of non‐genetic changes that alleviate the selective pressure confer long‐term advantage and may facilitate escape from extinction. Systematic analysis of outcomes as a function of population properties further identifies a non‐traditional regime of adaptation mediated by stochastic changes that are rapidly acquired within a lifetime. Contrasting model predictions with experimental findings shows that inheritance of dynamically acquired changes enables rapid adaptation to unforeseen challenges and can account for population dynamics that is either unexpected or beyond the scope of traditional models.

## Graphical abstract

A general model of inheriting changes that are acquired during the lifetime of individuals demonstrates tremendous benefits to the population, including escape from extinction in changing environments. Contrasting the model with experimental data shows that inheritance of acquired changes enables rapid adaptation to unforeseen challenges, and accounts for population dynamics that is unexpected or beyond the scope of traditional models.

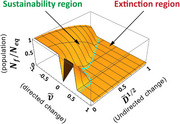

## 1 Introduction

Inheritance of non‐genetic changes and phenotypes that are acquired during the lifetime of individuals has been reported in cells, animals, and plants,^[^ ^1^, ^2^, ^3^, ^4^, ^5^, ^6^, ^7^ ^]^ including in response to environmental stressors (e.g., heat stress, endocrine disruptors, viral infection, nutrient limitation, changes in diet, toxic exposure, and traumatic experiences).^[^ ^8^, ^9^, ^10^, ^11^, ^12^, ^13^, ^14^, ^15^, ^16^, ^17^, ^18^, ^19^, ^20^, ^21^, ^22^, ^23^ ^]^ Followup studies have also identified distinct mechanisms supporting non‐Mendelian inheritance, such as acquisition of cellular memory by dynamic self‐reinforcement of gene expression,^[^ ^24^ ^]^ as well as by recovery of posttranslational modifications of nucleosomes after cell division,^[^ ^25^ ^]^ non coding RNA‐based acquisition of heritable resistance to viral infection in *Caenorhabditis elegans*,^[^ ^15^, ^16^ ^]^ DNA methylation‐based persistence of impacts of endocrine disruption in rats,^[^ ^10^, ^16^ ^]^ transgenerational modulation of Drosophila temperature response mediated by persistent histone modifications,^[^ ^8^, ^26^, ^27^ ^]^ heritable silencing of RNA in C. elegans, mediated by piRNA and chromatin components,^[^ ^28^ ^]^ inheritance of DNA methylation changes in heat‐exposed wild guinea pigs^[^ ^29^ ^]^ and hypoxia‐exposed fish,^[^ ^30^ ^]^ and inheritance of toxin‐induced phenotypes in Drosophila by altered deposition of maternal RNAs,^[^ ^31^ ^]^ as well as by persistence of changes in the gut microbiota.^[^ ^32^, ^33^ ^]^ Transgenerational phenomena may involve stochastic and/or deterministic changes and can be beneficial or non‐beneficial.^[^ ^34^ ^]^ Hallmark example of induced acquisition of beneficial changes that are transmitted to the offspring is provided by the acquisition of heritable resistance to viruses in *C. elegans*, which is, in turn, achieved by using the viral genome to mount a specific, RNA‐based defense that is transmitted across generations.^[^ ^11^ ^]^ Transmission of harmful effects has also been demonstrated, such as inheritance of metabolic dysregulation and susceptibility for obesity in response to altered nutrition and environmental toxicants.^[^ ^17^, ^35^ ^]^

Acquisition and inheritance of non‐genetic variations can modify the conditions of selection, leading to potentially unexpected impacts on population dynamics and persistence.^[^ ^36^, ^37^, ^38^, ^39^ ^]^ While the types of possible effects within and across generations are largely unknown, they may account for unexplained findings, including surprising escape from extinction in bacteria,^[^ ^40^ ^]^ acquisition of heritable adaptation to unforeseen conditions of stress in yeast^[^ ^41^, ^42^, ^43^ ^]^ and flies,^[^ ^44^ ^]^ and differential adaptation of epigenetically engineered strains of yeast.^[^ ^45^ ^]^ In addition to driving rapid adaptation, the inheritance of acquired changes may have long‐term impacts on the population, but it is not clear if and when they contribute to population sustainability, or alternatively, extinction. In general, these impacts depend on the magnitude, rate and type of environmental change,^[^ ^46^, ^47^, ^48^ ^]^ the properties of the species, and its population structure (manifested, for example, by kin phenotypic correlations^[^ ^49^ ^]^ and/or host‐symbiont interactions^[^ ^50^, ^51^ ^]^). This gives rise to a rich repertoire of possible outcomes, including cases in which the impacts turn from being beneficial to non‐beneficial and vice versa.^[^ ^34^, ^46^, ^52^ ^]^ The richness and complexity of possible scenarios presents a severe challenge to deciphering both short‐ and long‐term impacts of inheriting acquired changes. Addressing this challenge requires a modelling framework capable of drawing conclusions that are applicable to a wide range of populations and environmental regimes. An analogous problem of assessing impacts of complex, time‐varying ecology is addressed by population models.^[^ ^50^, ^51^, ^53^, ^54^, ^55^, ^56^, ^57^, ^58^, ^59^, ^60^, ^61^, ^62^, ^63^ ^]^ While enabling consideration of ecology‐driven changes in population structure on timescales that are smaller than one generation,^[^ ^58^, ^59^, ^64^ ^]^ these models do not account for inheritance of changes that are rapidly acquired (or made) by individuals. Initial evaluation of contributions of epigenetic inheritance under changing environments considered the transmission of epigenetic states that are assumed to be advantageous to the offspring.^[^ ^65^, ^66^ ^]^ In general, however, epigenetic variations are not necessarily beneficial. Moreover, since parents and offspring may be exposed to different environments, changes that were beneficial to the parents may be detrimental to the offspring. Comprehensive analysis of the effects of inheriting acquired changes therefore requires modeling that links changes in individuals to population impacts in different scenarios.^[^ ^67^ ^]^ Here, we introduce a population model of individuals undergoing dynamic changes that are either partially or fully transmitted to the offspring. This model is general enough to accommodate any type of stochastic or environmentally‐induced changes that are acquired within a lifetime and transmitted to offspring. This includes internal changes in individuals (e.g., epigenetic and symbiotic variations) as well as sustainable niche construction made by organisms. The model considers stochastic variations as well as environmentally‐induced responses that either reduce or increase the individuals' rate of death (weakening or strengthening the selective pressure).

## 2 Model Formulation

We consider an evolving population of *N* individuals, wherein the rates of birth and death of the *i*′*th* individual depend on its age *T*_*i*_(*t*) and state variable χ_*i*_(*t*) at time *t*. Assigning individuals with a dynamic variable χ_*i*_(*t*) whose value at the time of proliferation is transmitted to the offspring, allows us to link the state of individuals to population dynamics within and across generations. The state χ_*i*_(*t*) can represent any type of variable, phenotype, or process that is acquired and/or modified by individuals during their lifetime, and subsequently transmitted with some fidelity to the offspring. This includes internal states (e.g., changes in the epigenome and microbiome), as well as elements in the environment that are created and/or affected by the individuals (e.g. niche construction and cultural changes).

To derive a model for the time evolution of such a population, we extend the McKendrick–Von Foerster equation^[^ ^59^, ^68^, ^69^, ^70^ ^]^ by incorporating stochastic and directed (i.e., environmentally induced) changes in χ_*i*_ that are either perfectly or imperfectly transmitted to offspring. The time evolution of χ_*i*_ during a short period of time Δ*t* is given for the *i*′*th* organism by:

(1) $$\chi_{i}\left(t+\Delta t\right)=\chi_{i}\left(t\right)+f\left(\chi_{i},t\right)\Delta t+\sqrt{2D}\xi_{i}\left(t\right)$$

where the directed response in χ_*i*_ are governed by the function *f*(χ_*i*_, *t*), and ξ_*i*_(*t*) is a sequence of random numbers with zero mean and unit variance, representing diffusion in χ space with a “diffusion coefficient” *D*.

Applying standard methods of transforming stochastic equations for an individual to a probabilistic description at the population level (Section SI, Supporting Information), results in a differential equation for the time evolution of the population distribution *n*(*T*, χ, *t*), with population average state χ, rate of death *P*_*D*_(*T*, χ, *t*), as well as average directed response and diffusion terms.

(2) $$\begin{array}{ccc} \frac{\partial n\left(T,\chi ,t\right)}{\partial t} & & =-\frac{\partial n\left(T,\chi ,t\right)}{\partial T}-\frac{\partial }{\partial \chi }\left(f(\chi ,t)n(T,\chi ,t)\right)\\ & & +\;D\frac{\partial^{2}n\left(T,\chi ,t\right)}{\partial \chi^{2}}-P_{D}\left(T,\chi ,t\right)n\left(T,\chi ,t\right) \end{array}$$

(3) $$\begin{array}{cc} n(0,\chi ,t) & =\int \int d\chi^{\prime }dT^{\prime }P_{R}\left(T^{\prime },\chi^{\prime },t\right)I\left(\chi -\chi^{\prime }\right)n\left(T^{\prime },\chi^{\prime },t\right) \end{array}$$

(4) $$\begin{array}{cc} n(\infty ,\chi ,t) & =0 \end{array}$$

The rate of birth and fidelity of transmission are incorporated into the boundary condition at age *T* = 0 (Equation 3) and are specified, respectively, by *P*_*R*_(*T*, χ, *t*) and the inheritance function *I*(χ − χ′). Perfect inheritance corresponds to *I*(χ − χ′) = δ(χ − χ′), and imperfect inheritance is represented by a Gaussian function whose width determines the extent of deviation from perfect inheritance. Consideration of inheritance function that depends only on the distance of χ from χ′ is a crude representation of the complexity of replication, but it is nonetheless useful for recapitulating similarities between parents and offspring populations. The second condition (corresponding to *T* → ∞) states that no individual lives forever (Equation 4). It should be noted that this general formulation is equally valid for unicellular and multicellular organisms, as well as for different modes of reproduction and mechanisms of transmission.

The selective pressure on the population is governed by the dependencies of the rates of death and birth on the heritable state variable, χ. Since the distribution of χ in the population can change on timescales that are much faster than a single generation (e.g., in some cases of epigenetic, symbiotic and niche construction changes), individuals can modify the selective pressure by changing their state. To model changes that either reduce or increase the selective pressure on timescales that can be shorter than one generation, we consider a directed response function *f*(χ, *t*) that is proportional to the derivative of *P*_*D*_(χ, *t*) with respect to χ, namely:

(5) $$f\left(\chi ,t\right)=v\frac{\partial P_{D}\left(\chi ,t\right)}{\partial \chi }$$

where *v* is a directed response coefficient controlling the rate at which the change of χ increases or decreases the death rate. Accordingly, a negative *v* corresponds to a scenario in which individuals respond to selective pressure by changing χ in a direction that reduces the pressure (by decreasing the rate of death). A positive *v*, on the other hand, corresponds to changes in a direction that increases the selective pressure. Such a directed response function appears naturally in population models of slowly breeding host organisms that live in symbiosis with fast breeding bacteria (see Section SII, Supporting Information). In the case of directed response *f* that is independent of age (as in Equation 5), the rate of death depends only on χ and *t*.

Analytic solution of the time evolution of the population distribution is obtained by subjecting Equations ((2), (3), (4)) to combined, Fourier and Laplace transforms, applied to the state χ and age *T*, respectively, and solving the transformed equations using Hermite expansion (detailed procedures for solving Equations ((2), (3), (4)) are described in Section SIII (Supporting Information). Under conditions that are relevant for a wide range of observations and experimental scenarios, this permits derivation of an analytic solution in terms of exponential integral functions. The solution can then be compared with empirical data by: i) modifying the model's parameters and boundary conditions based on the experimental details, ii) plotting the time trajectories of the resulting population dynamics (e.g., using the Wolfram's *Mathematica* software), and iii) fitting normalized parameters (described in section C of the results) to measured features of the empirical population dynamics.

## 3 Results

### 3.1 Effects of Inheriting Acquired Changes in Stationary Environments

To investigate population dynamics in a stationary (time‐independent) environment, we consider a death rate that depends quadratically on χ and linearly on population size, *N*(*t*) = ∫∫dχ′d*T*′*n*(*T*′, χ′, *t*) (as in logistic growth):

(6) $$P_{D}\left(\chi \right)=aN\left(t\right)+b\chi^{2}$$

**Figure 1:**
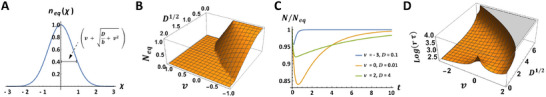
Properties of the equilibrium state in stationary environments and characteristic responses to transitions between environments. A) The equilibrium distribution *n*_*eq*_(χ) is a Gaussian function of χ with variance that depends on *D*, *v* and *b*. B) Equilibrium number of individuals *N*_*eq*_ as a function of *v* and *D* ^1/2^, for *r* = *a* = *b* = 1. C) Responses to a sudden shift between stationary environments, shown for the indicated sets of parameters. D) Log relaxation time τ following a shift Δχ = 1 for populations with the same *r* = 5 but different *v* and *D*. τ is measured in units of generation time 1/*r*. Note the divergence of the relaxation time in certain regions of the *v*, *D* parameter space. Visualizations of slices along v and D are available in Supplementary Figure S5.

The quadratic term represents a plausible approximation for the effect of environmental selection on the distribution of χ, at the vicinity of the value that minimizes the death rate. We first analyze the dynamics in the case of constant rate of reproduction *P*_*R*_(*T*, χ) = *r* and perfect inheritance *I*(χ − χ′) = δ(χ − χ′). The space of parameters in this case includes three population parameters (directed response *v*, diffusion *D* and replication rate *r*) and two environmental parameters (*a* and *b*) determining, respectively, the strength of resource limitation (“carrying capacity” ∝1/*a*) and the strength of the selective pressure on the distribution of χ. Despite the quadratic term in Equation (6), the time evolution of the population (Equations (2), (3), (4)) has an exact analytic solution for the distribution *n*(*T*, χ, *t*) (see derivation in Section SIII, Supporting Information). It shows that in stationary environments the population relaxes to a simple equilibrium distribution *n*_*eq*_(*T*, χ) = *rn*_*eq*_(χ)exp (− *rT*), exhibiting dependence on state *n*_*eq*_(χ) that reveals generic effects of the parameters *v*, *D*, *r*, *a* and *b* (**Figure** **1**A):

(7) $$n_{eq}\left(\chi \right)=\frac{N_{eq}}{\sqrt{v+\lambda^{2}}}\exp\left[-\frac{\chi^{2}}{2\left(v+\lambda^{2}\right)}\right]$$

The total population size at equilibrium is *N*_*eq*_ = [*r* − *b*(*v* + λ^2^)]/*a* (Figure 1B), with $\lambda^{2}=\sqrt{D/b+v^{2}}$ (derived in Section SIV, Supporting Information), specifying two contributions to the width of the equilibrium distribution, namely that of diffusion and that of directed response magnitude (regardless of its sign). The sign‐dependent contribution of the drift is, in turn, specified by *v*, so that the total variance at equilibrium is var(*n*_*eq*_) = λ^2^ + *v*. The dependence of *N*_*eq*_ and of *n*_*eq*_(χ), on the parameters *v* and *D* is summarized in Figures 1B and A respectively.

Note that *N*_*eq*_ decreases with *D* and increases with more negative *v*. Despite this benefit of more negative *v*, the equilibrium size of a population with constant *r* in a stationary environment is maximal when both *D* and *v* are zero (i.e., when *D* = 0, reduction of the selective pressure within a generation cannot increase the steady state size of the population beyond that of a population with *v* = 0). However, in any realistic scenario, *D* is larger than zero and *N*_*eq*_ increases as a function of the capacity to reduce the selective pressure within generation (negative *v*). Consideration of changes in more than one parameter shows that the same population *N*_*eq*_ can be maintained by different combinations of the parameters *v*,1 *D* and *r* (Figure 1B). For example, a given increase in *N*_*eq*_ due to reduction of selective pressure within generation (negative *v*) can also be achieved by faster replication (larger *r*) in populations of individuals that are incapable of reducing the selective pressure (i.e., having *v* = 0). Similarly, the negative impact of larger *D* can be counteracted either by faster replication (larger *r*) or by faster reduction of the selective pressure (more negative *v*). Thus, as long as the environment is constant, the same population size can be achieved by distinct “strategies”, corresponding, respectively, to altering the rate of replication and acquiring heritable changes that modify the selective pressure, either by directional changes (via *v*) or by stochastic variations (*D*).

### 3.2 Impacts of Inheriting Acquired Changes in Dynamic Environments

To investigate implications of inheritance of acquired changes under time‐varying (dynamic) environments, we consider shifts in the state χ_*min*_ that minimizes the death rate. For example, a sudden shift from χ_*min*_ = 0 in an old environment to χ_0_ in a new environment is defined by changing the death rate in Equation 6 to:

(8) $$P_{D}\left(T,\chi \right)=aN\left(t\right)+b{\left(\chi -\chi_{0}\right)}^{2}$$

For a population at equilibrium, the rates of death and birth are equal. The sudden shift in χ_*min*_ increases the death rate and drives dynamic changes in the distribution *n*(*T*, χ, *t*) until the population reaches a new equilibrium (Figure 1C). Since the rate of death *P*_*D*_(χ) and the resulting directed response *f*(χ) at equilibrium around χ = 0 in the old environment are the same as those around χ_0_ in the new environment, *N*_*eq*_ in the new environment is the same as in the old one, and the new equilibrium distribution *n*_*eq*_ is obtained by replacing χ in Equation (7) with χ − χ_0_. Parameter‐dependent analysis of populations that were at equilibrium in the old environment (Section SV, Supporting Information), reveals that the characteristic time for relaxation in the new environment (in units of 1/*r*) decreases with negative *v* (Figure 1D), indicating that relaxation is expedited by the ability to inherit acquired changes that reduce the selective pressure within generation (*v* < 0). On the other hand, the qualitative impact of stochastic changes (*D* > 0) as well as changes that increase the selective pressure (*v* > 0), depends on the magnitude of the changes (Figure 1D). If both *v* and *D* are sufficiently small, a larger *D*, or alternatively more positive *v*, contributes to expedited adaptation by broadening the distribution, thereby increasing the overlap between the tail of the initial distribution and the center of the new one. Beyond certain levels of *v* and *D*, however, further increase in either of them has the inverse effect of increasing the relaxation time, which diverges at finite values of positive *v* and *D* (Figure 1D).

In case of a single transition between stationary environments, the benefits of inheriting acquired changes that reduce the selective pressure are fully expected. However, if the environment continues to change, the inherited modifications in χ may be outdated and no longer advantageous. We therefore sought to investigate the potential long‐term impacts of inheriting acquired changes for different scenarios of change in the environment. For that, we considered two different types of dynamical change: a) periodic back and forth jumps in the selective pressure (Type I dynamics), and b) unidirectional jumps in the selective pressure (Type II dynamics). We incorporate these changes into the model by assigning χ_0_ with dynamical rules corresponding to the two types of change. Periodic jumps between states χ_0_ and −χ_0_ with inter‐jump Δ*t* are specified by:

(9) $$\chi_{0}\left(t+\Delta t\right)={\left(-1\right)}^{\Pi \left(t/\Delta t\right)}\chi_{0}\left(t\right)$$

where Π(*t*/Δ*t*) is a square wave function switching between 0 and 1 every Δ*t*.

Unidirectional jumps of size Δχ and inter‐jump duration Δ*t* are, in turn, specified by:

(10) $$\chi_{0}\left(t+\Delta t\right)=\chi_{0}\left(t\right)+\Delta \chi$$

In both cases, the magnitude and rate of change are determined by the additional environmental parameters Δχ and Δ*t*. The calculation of the final distributions for both types of dynamics is outlined in section VI of the SI.

**Figure 2:**
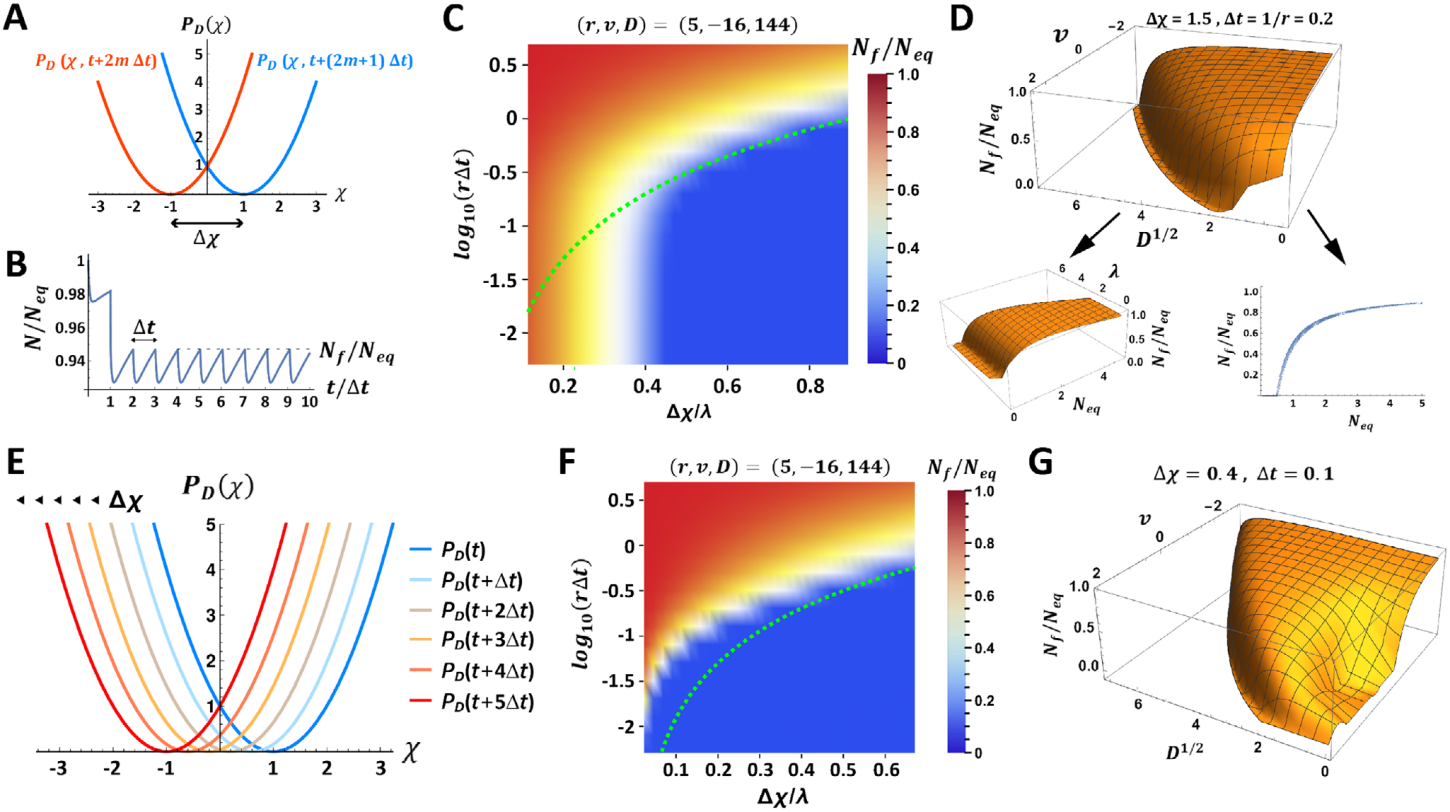
Population outcomes under exposure to dynamic environments. A) Periodic switching between two environments (Type I dynamics), represented by back‐and‐forth jumps (of magnitude Δχ=2) in the adaptive peak, with a duration of Δ*t* between consecutive jumps. B) Example of population recovery following a transition from a stationary environment to the periodically switching environment shown in (A). C) Normalized maximal level of the recovered population, *N*_*f*_/*N*_*eq*_, as a function of *r*Δ*t* and $\frac{\Delta \chi }{\lambda }$ for a specific choice of *r*, *v*, and *D*. Note the continuous transition from population survival to extinction in (Δχ, Δ*t*) space. Green dashed line displays the boundary of the necessary condition for survival, given in section VII of SI (below this line, the population goes extinct). D) Landscape of *N*_*f*_/*N*_*eq*_ as a function of *v* and *D* ^1/2^ for a given magnitude of the environmental switch (Δχ = 1.5) and duration between switches (Δ*t* = 0.2). Survival is supported by more negative values of *v* and smaller values of *D*. Lower panels: *N*_*f*_/*N*_*eq*_ vs. *N*_*eq*_ (right), and*N*_*f*_/*N*_*eq*_ as a function of *N*_*eq*_ and λ (left). E) Deteriorating environment (Type II dynamics), represented by unidirectional jumps of the adaptive peak by Δχ every Δ*t*. F) *N*_*f*_/*N*_*eq*_ as a function of *r*Δ*t* and $\frac{\Delta \chi }{\lambda }$ for the same choice of *r*, *v*, and *D* as in panel C. Green dash line displays the boundary of the necessary condition for survival given in Section SVII (Supporting Information). G) Landscape of *N*_*f*_/*N*_*eq*_ as a function of *v* and *D* ^1/2^ for successive jumps of magnitude Δχ = 0.4 and period Δ*t* = 0.1 between jumps. While negative *v* is generally beneficial (as in the case of periodic switching), the effects of positive *v* and *D* change from positive contribution to survival at small enough *v* and *D*, to deleterious impact at larger values of *v* and *D* (in contrast to the case of periodic switching).

To simplify the investigation of the effects of *v*, *D* and *r* in changing environments, we eliminated the dependence of the initial equilibrium population on these parameters by measuring population size *N*(*t*) in units of the initial *N*_*eq*_. We start by considering the case of periodically switching environment (**Figure** **2**A). Example of a typical trajectory of *N*(*t*)/*N*_*eq*_ for a surviving population in a switching environment is shown in Figure 2B. Each switch triggers a population decline (due to the sudden increase in selection pressure) followed by recovery that is mediated by a gradual shift of the distribution toward states of lower selective pressure. This process converges to periodic saw‐like pattern bounded by constant levels of minimal and maximal population size. The maximal level of the recovered population, *N*_*f*_, is a measure of how resilient the population is to the environmental perturbation.

Analysis of *N*_*f*_/*N*_*eq*_ as a function of Δχ and Δ*t*, reveals a sharp separation between survival and extinction (Figure 2C). A similar separation is noted by plotting *N*_*f*_/*N*_*eq*_ as a function of *v* and *D* ^1/2^ for a specific choice of Δχ and Δ*t* (Figure 2D), demonstrating that the recovered population decreases with larger *D* and increases with more negative *v*. Since the magnitude of the directed response is proportional to both *v* and χ, negative *v* confers two benefits: shifting the distribution towards the adaptive peak and narrowing it around the peak (Figure S5, Supporting Information). A sufficiently negative *v* also enables sustainability of populations that would otherwise go extinct (Figure 2D and Section SVII, Supporting Information). Plotting *N*_*f*_/*N*_*eq*_ vs. *N*_*eq*_ and λ at fixed Δχ and Δ*t* (Figure 2D, bottom left panel), shows that *N*_*f*_ is fully determined by the initial equilibrium size of the population, *N*_*eq*_ and by the value of λ. For a sufficiently small Δ*t*, *N*_*f*_ is largely determined by the initial equilibrium size, *N*_*eq*_ and is only weakly dependent on λ (Figure 2D, bottom right and left panels).

In the case of Type II dynamics (Figure 2E), the state that minimizes the selective pressure, χ_0_(*t*), undergoes unidirectional shifts every Δ*t* by an amount Δχ. Despite this successive deterioration of the environment, for every Δχ and Δ*t*, there is a range of parameters *v*, *D*, *r*, *a* and *b*, supporting a non‐vanishing value of *N*_*f*_. For the most part, the effects of the environmental and population parameters are qualitatively similar in deteriorating and periodically switching environments (compare Figures 2F and 2C). However, in contrast to periodically switching environment, in the case of deteriorating environment, populations with small enough *D* may benefit from slow acquisition of changes that increase the selective pressure (demonstrated in Figure 2G by the increase of *N*_*f*_/*N*_*eq*_ with positive *v* as long as it is small enough). This benefit stems from the contribution of positive *v* to the broadening of the distribution, enabling survival of individuals with a state that is sufficiently close to χ_0_(*t*) (analytic expressions for survival and extinction are derived in Section VII, Supporting Information).

### 3.3 Parameter Rescaling Simplifies the Analysis, Enabling Model Extensions to More Complex Scenarios

We have thus far analyzed influences of changes in the environment on populations with a given set of properties, focusing on reproduction rate, *r*, the extent of acquiring stochastic changes, *D*, and the magnitude/type of directed response, *v*. While providing a simple representation of rather complex phenomena, the combined sets of population and environmental properties still comprise a large parameter space that complicates the analysis and conclusions. This becomes even more challenging when the model is extended to consider additional features, such as imperfect inheritance and age‐dependent decline in fertility. We therefore sought to simplify the analysis by reducing the model to a smaller set of effective parameters, such that a given set represents a large variety of more complex scenarios.

We found that the entire dynamics can be fully represented by two population properties (“responsiveness” $\tilde{v}$ and“diffusivity” $\tilde{D}$), and re‐scaled measures of the environmental challenge $\Delta \tilde{\chi }$, population size $\tilde{N}$, and time $\tilde{t}$ (see Section SVIII, Supporting Information, for derivation):

(11) $$\tilde{v}=\frac{vb}{r};\;\tilde{D}=\frac{bD}{r^{2}};\;\Delta \tilde{\chi }=\frac{\Delta \chi }{v+\lambda^{2}};\;\tilde{N}=\frac{N}{N_{eq}};\;\tilde{t}=tr$$

**Figure 3:**
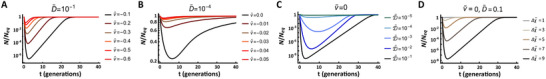
Reduced representation of population dynamics. A) The impact on the population *N*/*N*_*eq*_ due to a single shift in environment $\Delta \tilde{\chi }=10$ is plotted against generation time *rt*, for $\tilde{D}=10^{-1}$ and different values of $\tilde{v}$. B) Same as (A) for $\tilde{D}=10^{-4}$. C) Same for $\tilde{v}=0$ and different values of $\tilde{D}$. D) *N*/*N*_*eq*_ vs *rt* for different values of $\Delta \tilde{\chi }$, $\tilde{D}=0.1$ and $\tilde{v}=0$.

The responsiveness and diffusivity correspond to dimensionless parameters representing the directed response *v* and diffusion *D*, in units of *r*/*b* and *r* ^2^/*b*, respectively. The re‐scaled environmental challenge is a dimensionless parameter that provides an intrinsic measure of challenge strength by measuring the deviation from the adaptive peak Δχ in (population‐specific) units of distribution width *v* + λ^2^. The remaining measures represent population size and time in units of the equilibrium size, *N*_*eq*_, and reproduction time, 1/*r*. The practical meaning of this is that populations with identical $\tilde{v}$ and $\tilde{D}$ that experience the same $\Delta \tilde{\chi }$ will exhibit indistinguishable time evolution $\tilde{N}\left(\tilde{t}\right)$. The entire space of population dynamics is therefore captured by three dimensionless parameters, $\tilde{v}$, $\tilde{D}$, and $\Delta \tilde{\chi }$. In the case of a sudden environmental shift, for example, the repertoire of population trajectories is fully characterized by plotting $\tilde{N}$ vs. $\tilde{t}$ for different values of $\tilde{D}$, $\tilde{v}$ and $\Delta \tilde{\chi }$ (**Figure** **3**). It shows that the initial population drop and the time for complete recovery are both decreasing non‐linearly as a function of negative $\tilde{v}$ (Figure 3A), but the shape of the population trajectory is also dependent on the value of $\tilde{D}$ (compare Figures 3B and 3A). The effect of changing $\tilde{D}$ is less intuitive (larger $\tilde{D}$ in Figure 3C is associated with smaller population drop and faster recovery) because the strength of the rescaled challenge $\Delta \tilde{\chi }$ is measured in units of population width. Since a larger $\tilde{D}$ corresponds to a broader distribution, increasing $\tilde{D}$ at a fixed $\Delta \tilde{\chi }$ means that the strength of the non‐scaled challenge, Δχ, is also increasing. Moreover, since the re‐scaled challenge is measured in population‐specific units of distribution width, the reduction in parameter space comes at the expense of clear separation between environmental and population parameters. And yet, in addition to simplifying the analysis, the reduced space proves to be useful for distinguishing dynamical profiles of possible population trajectories (as demonstrated in the recapitulation of the experimental findings in Section SVIII, Supporting Information).

We used this re‐scaled representation to investigate how the impacts of transmitting acquired changes are affected by age‐dependent fertility and imperfect inheritance. For simplicity, we considered individuals whose rate of reproduction declines exponentially with age and whose state is imperfectly transmitted to the next generation (in Section SX, Supporting Information, we show that more realistic age‐dependent fertility leads to similar conclusions). These features were incorporated by re‐defining the reproduction rate *P*_*R*_(*T*) and inheritance function *I*(χ − χ′) as follows:

(12) $$P_{R}\left(T\right)=re^{-RT}$$

(13) $$I\left(\chi -\chi^{\prime }\right)=\frac{1}{\sqrt{2\pi }\sigma }e^{-{\left(\chi -\chi^{\prime }\right)}^{2}/2\sigma^{2}}$$

where *R* is the rate of fertility decline and σ measures the deviation from perfect inheritance. For *r* > *R*, the average lifetime 〈*T*〉 in a population with age‐dependent exponential decline in fertility is proportional to 1/(*r* − *R*) (Section SVIII, Supporting Information). Populations with larger *R* therefore consist of longer‐lived individuals whose reproductive phase extends over a smaller fraction of their lifespan. The inheritance function describes the probability that a parent with state χ′ gives rise to offspring with state χ. The difference between these states can be caused, for example, by epi‐mutations encountered in the process of replication (“imperfect replication”). As shown in Section SVIII (Supporting Information), the solutions of a model with these complications are readily obtained from those of the simpler model by shifting the parameters *r* and *D* in the expressions for *n*_*eq*_ and *N*_*eq*_: *r* → *r* − *R* and *D* → *D* + *r*σ^2^/2, respectively. Since the time evolution (in units of *t*_*c*_ = 1/(*r* − *R*)) is indistinguishable for populations that have the same $\tilde{v}$ and $\tilde{D}$ (Figure S6A, Supporting Information), the solutions for the case of declining fertility and imperfect inheritance are obtained from those of *R* = 0 and σ = 0 by using the shifted parameters in the re‐scaling (Equation S78, Supporting Information). In terms of the rescaled parameters, the equilibrium population in a stationary environment is given by:

(14) $$N_{eq}\;=\tilde{N}\left(1-\tilde{v}-\sqrt{{\tilde{v}}^{2}+\tilde{D}}\right)$$

Impacts of changing *r*, *R*, *v*, *D* and σ on *N*_*f*_/*N*_*eq*_ are then deduced from Figure S6B in SI, by determining the corresponding values of the dimensionless parameters $\tilde{v}$ and $\tilde{D}$. Effects on the absolute size *N*_*f*_ (without normalization by *N*_*eq*_) are subsequently determined by taking into account the dependence of *N*_*eq*_ on $\tilde{v}$ and $\tilde{D}$ (Equation 14).

### 3.4 Reconstructing and Interpreting Rapid Adaptation of Real Populations

The generality of the model facilitates its application to a wide range of adaptation scenarios by making simple adjustments of the model in accordance with the details of the population and environmental conditions. Since suitable examples of long‐term dynamics in experimental populations of multicellular organisms are yet to be generated, and the model does not distinguish between multicellular and unicellular populations, we tested if the model can reproduce empirical unicellular population dynamics that is either unexpected or has not yet been modeled. We applied this to distinct scenarios of adaptation to a stressful environment, namely: 1) Bacterial adaptation to a joint regimen of toxic exposure and high frequency population dilutions that was expected to result in extinction,^[^ ^40^ ^]^ 2) Adaptation of yeast to unforeseen stress in a chemostat,^[^ ^42^ ^]^ and 3) Yeast adaptation that is experimentally modified by epigenetic gene silencing.^[^ ^45^ ^]^ In all three cases, the transition to a stressful environment is followed by rapid drop of the population and subsequent recovery on unexpectedly fast timescales. To simplify the reconstruction of the experimental results using the model, we first determine which values of the reduced variables $\left(\tilde{v},\tilde{D},\Delta \tilde{\chi }\right)$ fit the empirical population dynamics most accurately; any combination of parameters (*r*, *v*, *D*, *a*, *b*, Δχ) that yields the same values of $\tilde{v},\tilde{D}$, and $\Delta \tilde{\chi }$ would map onto the same curve (when measured in generation time and *N*/*N*_*eq*_). We then set *r* = 1 (*r* = 1/generation time) and compute the values of other parameters using Equation 11. The case of a single shift admits an analytic solution for the population dynamics as a function of the reduced parameters (Section SIX, Supporting Information), enabling rigorous evaluation of the uniqueness of mapping the experimental population dynamics to the reduced variables, and the sensitivity of this mapping to parameter changes (Figure S8, Supporting Information).

**Figure 4:**
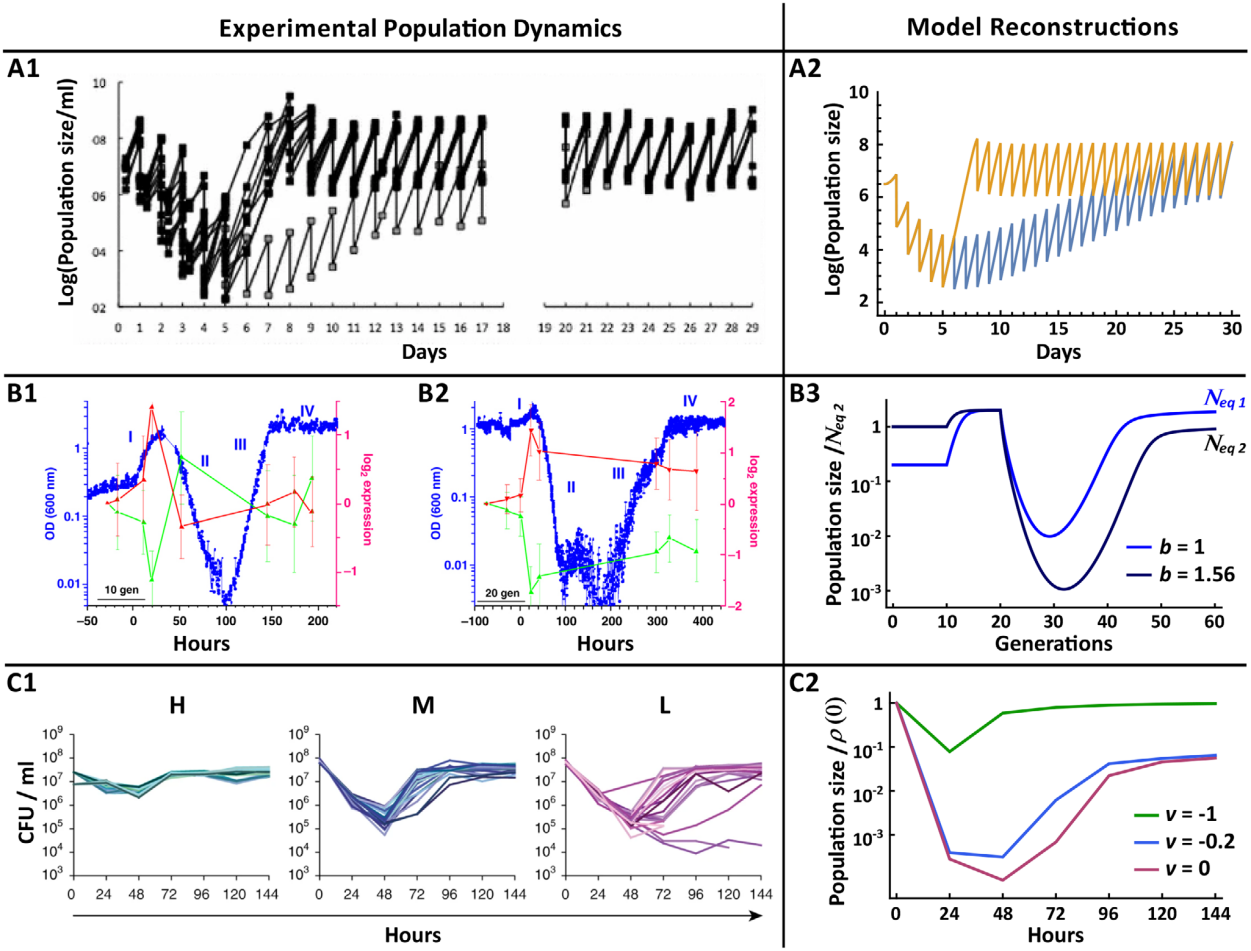
Comparison between in vivo trajectories of adaptation in 3 different experiments and model predictions. A1) Batch culture experiments of bacterial population that is challenged for growth on a toxic carbon source (biphenyl) and is subjected to sudden 100‐fold dilutions every 24hrs.^[^ ^40^ ^]^ Shown are population trajectories in experimental replicates in which the dilutions (indicated by sudden population drops) were suspended between days 4 and 7, except for one case (gray trajectory) with dilutions that were uninterrupted throughout the experiment. A2) Model reconstruction of the large population drop in the first 5 days and the subsequent escape from extinction timescale with and without suspension of the dilutions (orange and blue trajectories, respectively). Model trajectories were generated with the following parameters: *r* = 1, *a* = 1, *b* = 1, Δχ = 10, *v* = −0.0097, *D* = 2.4 · 10^−4^. B1) Adaptation of yeast (*S. cerevisiae*) to an unforeseen challenge in a chemostat (reproduced from Stern et al.^[^ ^42^ ^]^). The population was genetically rewired so as to recruit a gene that is essential for histidine production (*HIS3*) to the (unrelated) GAL4 promoter that is shut off by switching from galactose to glucose media (Phase I). The strong repression of the *GAL4:HIS3* gene in histidine‐free media results in histidine depletion, leading to arrested growth and population collapse in phase II. This is then followed by remarkable recovery on a similar timescale as the collapse. B2) Same as (B1) with aggravation of the challenge by inhibition of histidine by treatment with 3‐AT. B3) Reconstruction of the stage‐dependent features of the population dynamics in B1 (blue curve) and B2 (black), using a model based exclusively on stochastic changes (*v* = 0) acquired during a lifetime: *r* = 1, Δχ = 2.496, *D* = 5.6 · 10^−4^, *b* as indicated in the figure, *a* = 1 and *a* = 5 in galactose medium (*t* < 10), with and without 3‐AT, respectively, *a* = 0.5 and 1 in glucose medium prior(0 < *t* < 20) and after (*t* > 20) the development of histidine deficiency, respectively. C1) Dynamics of yeast populations engineered with of high (H), medium (M), and low(L) degree of heritable silencing of *URA3* and exposed in uracil‐free medium to 5‐fluoroorotic acid (5‐FOA) which is toxic for cells that express the *URA3* gene. Reproduced from Stajic et al.^[^ ^45^ ^]^ (C2). Reconstruction of the population dynamics with various degree of heritable silencing of *URA3* by a model with variable degree of directed response *v* that reduces the selective pressure within a generation. *r* = 1, *a* = 1, *b* = 0.01, Δχ = 12, *D* = 0.001, and the respective *v* values are indicated in the figure. Panel A1 reproduced from Travisano et al.^[^ ^40^ ^]^ under creative commons license CC BY 4.0. Panel B1 and B2 reproduced from Stern et al.^[^ ^42^ ^]^ under creative commons license CC BY 4.0. Panel C1 reproduced with permission from Stajic et al.^[^ ^45^ ^]^ The rights to panel C1 are retained by Nature Publishing Group.

In Travisano et al.^[^ ^40^ ^]^ (**Figure** **4** A1), populations of soil bacteria (*Alcaligenes xylosoxydans*) were grown in liquid culture in media that contains a toxic substance (biphenyl) as a sole carbon source, and propagated by serial, 100‐fold dilutions with a constant time between dilutions. Following a single dilution in biphenyl medium, the population recovers within about 2 days.^[^ ^40^ ^]^ Successive dilutions at a shorter interval (e.g., 1 day) were therefore expected to result in consecutive population declines and eventual population extinction. Surprisingly, however, the expected decline was observed only during the first 5 dilution cycles, following which the population recovered within about 15 days to a stationary state of repeated cycles of decline and growth (Figure 4 A1). Notably, the same stationary state was observed in independent experiments, with and without transient suspension of the dilution regimen between days 5 and 7 (with the former case resulting in faster recovery to the stationary state). To test if our model can capture these features, we represented the toxic challenge of biphenyl by introducing a shift Δχ at *t* = 0, and incorporated the dilutions by 100‐fold reduction of population size once a day. The resulting model formulation was indeed able to account for the experimental features of Figure 4 A1, including the escape from extinction, the scale of population decline, the timescale of recovery, and the stationary cycles of population decline and growth (Figure 4 A2).

The case of yeast adaptation to unforeseen stress^[^ ^42^ ^]^ involved genetic rewiring of the cells, so as to shift the *HIS3* gene (essential for histidine synthesis) from its endogenous regulation to ectopic regulation by the GAL system (the native *HIS3* was substituted with *HIS3* under a GAL4 promoter). The rewired cells were maintained in a chemostat fed with galactose medium lacking histidine. Upon switching from galactose to glucose (phase I in Figure 4 B1), the growth is initially increasing due to the utilization of a preferred carbon (glucose) but the repression of the GAL4 promoter leads to depletion of the histidine pool that was produced under galactose. Subsequent shortage of histidine (phase II) creates a severe challenge manifested by exponential drop of the population to very low levels. Since the production of histidine by the rewired cells is exclusively dependent on regulation that was evolutionary shaped for repression under glucose, the cells cannot rely on an existing program of histidine synthesis in the presence of glucose. Despite the initial lack of program for histidine production, the initial population collapse is arrested within 10–20 generations, following which the population recovers (phase III) to the typical population size under glucose (phase IV). In another realization of this experiment, the challenge of overcoming the histidine shortage was further aggravated by supplementing the media with the *HIS3* inhibitor, 3‐AT, leading to a larger population drop and delayed recovery compared to adaptation without 3‐AT (Figure 4 B2 vs B1). Prior to the switch, on the other hand, the GAL4 promoter drives overproduction of *HIS3*, causing a metabolic burden that is alleviated by 3‐AT. This effect is manifested by a larger population size in galactose + 3‐AT media vs. galactose alone (compare initial levels in Figure 4 B2 vs B1).

To test if the model can account for the outcomes of these experiments, we adjusted it so as to incorporate: i) the deleterious effect of *HIS3* overproduction driven by GAL4 under galactose, ii) the transition from galactose to (more efficient) glucose metabolism, iii) the severe challenge of histidine deficiency due to the silencing of the GAL4 promoter under glucose, and iv) aggravation of this challenge by further inhibition of *HIS3* in the presence of 3‐AT. Since the shift from galactose to glucose corresponds to an effective increase in “carrying capacity”, 1/*a* (independent of the challenge of histidine deficiency), we accounted for it by lowering *a* at the time of transition (beginning of phase I, *t* = 10 generations). The challenge of histidine deficiency was modelled by shifting the state of minimal death rate (adaptive peak). This was implemented by Δχ that was introduced at the end of phase I (*t* = 20 generations).^[^ ^71^ ^]^ Lastly, the positive effect of *HIS3* inhibition in galactose media (alleviation of histidine overproduction) and its negative impact under glucose (aggravation of the challenge) were implemented, respectively, by imposing a higher carrying capacity (smaller *a*) under galactose with 3‐AT (vs. no 3‐AT) at 0 < *t* < 10 generations, and placing a stronger penalty on deviations from the adaptive peak (larger *b*). Incorporating these experimental details into the model allowed it to capture all the specific features of the population dynamics in all stages of the experiment, with and without supplementation of 3‐AT, as demonstrated in Figure 4 B3. Most strikingly, we found that the dynamic profile of population decline and recovery in Figures 4 B1 and B2 is incompatible with a directed response that reduces the individual's death rate (negative *v*) but is, however, nicely captured by stochastic accumulation of changes within a lifetime of individuals (non‐vanishing *D*) followed by transmission to the offspring (Figure 4 B3). Accounting for this population dynamics without relying on a pre‐programmed response (i.e., *v* = 0) demonstrates the feasibility of reducing the selective pressure and restoring cell growth in a novel environment, by stochastic (and sufficiently rapid) acquisition of heritable cell states.

Incompatibility with deterministic induction of an adaptive response is indicated by the repertoire of possible solutions (in Figure 3A,B): for *v* < 0 there are no trajectories corresponding to population collapse by 2‐3 orders of magnitude that extends for 10 generations and is followed by a complete recovery within 10‐20 generations. The ability to account for these observations by acquisition and transmission of stochastic changes in the absence of a pre‐programmed response, is remarkably consistent with lack of prior regulation that specifies expression of the *GAL4‐HIS3* gene in glucose medium.

The study of Stajic et al.^[^ ^45^ ^]^ provides an opportunity to contrast the model with an experiment that was specifically designed to investigate potential evolutionary impacts of epigenetic gene silencing. For that, they translocated the uracil biosynthesis gene, *URA3*, across subtelomeric regions of the chromosome so as to generate distinct populations of yeast, each with a different degree of heritable silencing of *URA3*. The expression of *URA3* is required for growth in uracil‐free media but induces cell death in the presence of 5‐fluoroorotic acid (5‐FOA). Tuning the degree of *URA3* silencing therefore modulates the extent to which these populations can overcome toxic exposure to 5‐FOA by switching off the expression of *URA3*. Dynamic profiles of adaptation to 5‐FOA are displayed in Figure 4 C1 for populations that were engineered with of high (H), medium (M) and low(L) degree of heritable silencing of *URA3*. The different rates of heritable *URA3* silencing under challenge with 5‐FOA are represented in our model, by large, intermediate, and low magnitude of negative directed response, *v*. The larger the magnitude of negative *v*, the faster the silencing of *URA3* in response to 5‐FOA challenge. Figure 4 C2 demonstrates that a model with population‐specific *v* and growth rate *r* of 16 generations per day, suffices to reproduce the population dynamics of the three experimental populations.

Taken together, these results demonstrate that qualitative features of different kinds of experiments on different organisms can be well represented by our model, despite its apparent simplicity. Full quantitative fitting can be performed if more details about individual experiments are introduced into the model.

## 4 Discussion

In contrast to rare genetic mutations that can be transmitted over many generations, non‐genetic changes are frequently acquired within a lifetime but are generally less stable than genetic variations. Rapidly acquired changes include stochastic variations and preprogrammed reactions to the environment that could potentially reduce or increase the rate of death (and/or reproduction) of individuals. Predicting concrete impacts of inheriting these changes, is however, confounded by population‐ and environmental‐specific effectors, including the population properties, structure and state, and the type, magnitude and dynamics of environmental changes. Here, we showed that the scope of short‐ and long‐term impacts of dynamic acquisition and inheritance of non‐genetic changes can be investigated using a simple population model that can be readily adjusted to predict challenging empirical outcomes. This modelling links arbitrary changes in the state of individuals to population dynamics within and across generations, by assigning individuals with a dynamic variable χ_*i*_(*t*) whose value at the time of proliferation is transmitted to the offspring. This variable can represent internal states (e.g., changes in the epigenome and microbiome) as well as external structures that are created and/or modified by actions of individuals (e.g., niche construction). To include environmental induction of adaptive responses as well as maladaptive reactions, we considered a model in which: i) the death rate is a function of χ_*i*_, and ii) the rate of directional change (directed response) *f*(χ_*i*_) is proportional to the gradient of the death rate function, $f\left(\chi_{i}\right)=v_{i}\frac{\partial P_{D}\left(\chi_{i},T\right)}{\partial \chi_{i}}$. To capture population‐level dynamics, we replaced individual‐specific changes with population average directed response and diffusion, thus enabling derivation of an equation for the time evolution of the population distribution *n*(χ, *T*, *t*), subject to state‐, age‐ and time‐dependent rates of death *P*_*D*_(χ, *T*, *t*) and reproduction *P*_*R*_(χ, *T*, *t*). Depending on the sign of *v*, it represents adaptive responses that were selected in previous generations, or alternatively, maladaptive reactions (e.g., non‐beneficial by‐products of mechanisms that are not relevant to the current environment). In most of this work, we considered directed response that affects the rate of death. It is important to note, however, that the same drift term can also represent environmental increase or decrease in reproduction rate, *r*. All the stochastic changes that are acquired within a lifetime and transmitted across generations are represented by the diffusion term. The impact of these changes depends on the population properties and environmental context. For the most part, they have a negative impact, but in some cases they can also be beneficial and even essential when the population is not equipped with a sufficiently effective response to a challenging environment (e.g., when *v* ⩾ 0). While the acquisition and inheritance of stochastic changes (represented by *D*) is not as beneficial as deterministic reduction of death rate within a generation (a dircted response with negative *v*), it has the unique advantage of facilitating dynamic acquisition of beneficial changes within and across generations.^[^ ^72^ ^]^ As such, it extends the validity of the main insights of the model to the case of inherited adaptations that are newly forming during the individual's lifetime (as opposed to adaptive responses that were selected in previous generations).^[^ ^72^, ^73^ ^]^

The time evolution of the population distribution is fully determined up to the time in which one of the population parameters (*r*, *R*, *D*, *v*, and σ) undergoes a significant change. Assuming that this time is similar to the timescale for acquiring genetic changes that drive noticeable effects at the population‐level (typically over 10^3^ generations^[^ ^74^ ^]^), the model covers durations extending from immediate changes to many generations. This enables deciphering of short‐ and long‐term impacts of heritable changes that are acquired within every lifetime. Since the timescales for shifting and broadening the distribution are (*b*(|*v*|))^−1^ and ${\left(4b\lambda^{2}\right)}^{-1}={\left(4b\sqrt{\left(v^{2}+D/b\right)}\right)}^{-1}$, respectively, the multi‐generational impacts of acquiring heritable changes are determined by choosing parameters for which one of these timescales is longer than 1/*r*.

Under deteriorating environments, the acquisition of heritable changes that shift the distribution toward the adaptive peak (*v* < 0) confers two obvious benefits: reduction of death rate within a generation, and persistence of the lower death rate in newborns. In the case of periodic switching, however, the effect of the transmission depends on the time of environmental switching and is not necessarily beneficial. For switching time Δ*t* larger than a few generations, the transmission is mostly beneficial (as noted by Lachmann and Jablonka^[^ ^66^ ^]^). Conversely, when Δ*t* is comparable to the generation time, newborns often inherit χ that is farther away from the new adaptive peak, thus contributing to an initially elevated rate of death. Nonetheless, when *v* is negative, the deleterious impact of transmitting a maladaptive state is counteracted by subsequent changes that reduce the death rate within a generation. Sufficiently negative *v* also enables escape from extinction within a wide range of environmental challenges. While escape from extinction can also be achieved by faster reproduction (larger *r*), the rate of reproduction cannot be arbitrarily large without compromising survival (increasing *P*_*D*_) and lowering the accuracy of reproduction (increasing σ), thus limiting the rescue from extinction to a restricted range of *r*.

Consideration of heritable stochastic variations has been traditionally limited to the case of rare mutations with no distinction of when they are generated. Extending this to variations that are acquired within a lifetime and transmitted with different degrees of fidelity, identified a significant distinction between variations that are encountered prior and during reproduction (represented, respectively, by *D* and σ). While both are typically deleterious, the negative effect of variations during reproduction scales with the reproduction rate, thus providing advantage for higher fidelity of transmission. Realizing this advantage in multicellular organisms is limited by the mechanistic difficulty of transmitting acquired changes across the germline. However, cases of easy transmission (e.g., inheritance of cytoplasmic factors in unicellular organisms, inheritance of gut microbiota in animals, long‐lasting niche constructions and cultural inheritance) are typically associated with high fidelity of inheritance.

The applicability and explanatory power of this model were assessed, respectively, by evaluating the feasibility of incorporating relevant details of adaptation of real populations, and the ability to account for experimental findings that are either not covered or appear to deviate from expectations based on genetic models of adaptation. We showed that the model can be easily adjusted to accommodate diverse experimental conditions and adaptation scenarios, including epigenetic manipulation of the ability to cope with maladaptive conditions,^[^ ^45^ ^]^ overcoming an unforeseen challenge without a pre‐existing regulation that guides the adaption,^[^ ^42^ ^]^ and escape from extinction under the combined challenge of frequent dilutions of a population that grows on a toxic carbon source.^[^ ^40^ ^]^ Moreover, by incorporating the relevant details of each experiment, we found that our model formulation is equally applicable to overcoming environmental suppression of growth rate (as opposed to increase of death rate), as well as to adaptation in a chemostat. The success of reproducing the surprising findings of these complex experiments, demonstrates how a general (and seemingly simple) model formulation provides sufficient flexibility to incorporate specific experimental and biological details which enable it to generate predictions that are in remarkable agreement with empirical data. Perhaps the most notable insight of the comparison with experimental results was provided by the case of adaptation to an unforeseen challenge. In agreement with the experimental design that invalidated reliance on pre‐existing adaptation to the challenge, we found that the shape of the adaptation trajectory is compatible with non‐genetic acquisition (and inheritance) of stochastic changes (corresponding to the diffusion term), but not with a directional response that alleviates the challenge (corresponding to directed response with negative *v*). While, in principle, our model can also be taken to represent the time evolution of populations undergoing genetic changes, this case would be characterized by $\tilde{v}=0$ and $\tilde{D}$ that is several orders of magnitude smaller compared to the case of non‐genetic changes. In yeast, for example, the observed rate of single nucleotide mutations (SNMs) is 1.67 · 10^−10^ per base per generation.^[^ ^75^ ^]^ Stochastic variations in χ that are exclusively caused by SNMs in a specific genomic locus would therefore translate in our model to *r* = 1[*gen* ^−1^] and $D=\tilde{D}=1.67\cdot 10^{-10}$. Even if we assume that a rescuing change in χ can be achieved by a mutation in any of 10^3^ independent genomic positions, the respective *D* would still be 3 orders of magnitude smaller than the value that fits the experimental population dynamics in Figure 4B (*D* = 5.6 · 10^−4^).

Altogether, we showed that a model that is simple enough to admit analytic solutions can nonetheless generate broadly applicable insights about the short‐ and long‐term impacts of acquisition and transmission of non‐genetic changes, and proves to be remarkably effective in predicting and interpreting experimental findings that exceed the scope of possible outcomes of genetic adaptation by selection of rare mutations. Moreover, the proposed modeling framework is readily extendable to arbitrary dependencies of the rates of death and reproduction on age, state, and time (examples of state‐dependent rate of reproduction as well as its non‐monotonic dependence on age are provided, respectively, in Sections SIX and SX, Supporting Information). It should be noted, however, that extensions of the model to arbitrary functions of death and birth rates are typically not amenable to analytic solutions, but can be thoroughly investigated numerically. While the outcomes in these cases are generally dependent on the specific dependencies of the death and birth rates, the inheritance of non‐genetic changes that are acquired within a lifetime is still expected to have long‐term effects in a wide range of formulations.

## Conflict of Interest

The authors declare no conflict of interest.

## Supporting information

Supporting Information

## Data Availability

The data that support the findings of this study are available in the supplementary material of this article.
